# Reelin Deficiency and Synaptic Impairment in the Adolescent Prefrontal Cortex Following Initial Synthetic Cannabinoid Exposure

**DOI:** 10.1016/j.bpsgos.2024.100426

**Published:** 2024-11-28

**Authors:** Thenzing J. Silva-Hurtado, Gabriele Giua, Olivier Lassalle, Leila Makrini-Maleville, Benjamin Strauss, Jim Wager-Miller, Jean-Marc Freyermuth, Ken Mackie, Emmanuel Valjent, Olivier J.J. Manzoni, Pascale Chavis

**Affiliations:** a Aix-Marseille University, INSERM, INMED, Marseille, France; b Cannalab Cannabinoids Neuroscience Research International Associated Laboratory, INSERM, Aix-Marseille University, Marseille, France and Indiana University, Bloomington, Indiana; c Institute of Functional Genomics, University of Montpellier, INSERM, CNRS, Montpellier, France; d The Gill Institute for Neuroscience and Department of Psychological and Brain Sciences, Indiana University, Bloomington, Indiana; e Aix-Marseille University, CNRS, I2M, Marseille, France

**Keywords:** CB1R, Initial exposure, Prefrontal cortex, Reelin, Synaptic plasticity, Synthetic cannabinoid

## Abstract

**Background:**

Adolescent cannabinoid exposure can have long-lasting effects on the brain, particularly in the prefrontal cortex, where the reelin protein plays a crucial role in neural organization. Chronic cannabinoid exposure leads to reelin deficiency and behavioral abnormalities, but the underlying mechanisms remain unclear. With the increasing use of synthetic cannabinoids (SCs) among young people, understanding these effects is crucial.

**Methods:**

We examined the cellular and synaptic consequences of initial SC exposure in adolescent male mice 1 day after a single in vivo exposure to WIN 55,212-2. Our approach combined immunohistochemistry, Western blots, conditional CB_1_ receptor (CB1R) knockout mouse lines, quantitative polymerase chain reaction, and ex vivo electrophysiology to investigate the effects of SC on reelin expression and synaptic plasticity. Additionally, single-molecule fluorescent in situ hybridization profiling was used to identify cellular coexpression patterns of reelin and CB1Rs.

**Results:**

Our findings indicate that a single exposure to SC decreased reelin expression in specific prefrontal cortex layers accompanied by disrupted proteolytic fragmentation but not changes in messenger RNA expression. Single-molecule fluorescent in situ hybridization profiling revealed a strong coexpression of CB1R and reelin. Furthermore, our pharmacological and genetic approaches demonstrated that CB1Rs in GABAergic (gamma-aminobutyric acidergic) neurons mediate the SC-induced decrease in reelin. This decrease in reelin results in a reduction in long-term potentiation, phenocopying reelin haploinsufficient mice. Notably, we restored long-term potentiation by infusing reelin bilaterally, establishing a functional link between reelin depletion and synaptic deficits.

**Conclusions:**

These findings provide new insights into the neural consequences of adolescent cannabinoid consumption and highlight the critical role of reelin in the cellular mechanisms associated with SC initiation during adolescence.

## Plain Language Summary

Recreational use of synthetic cannabinoids (SCs) is popular among adolescents, but their effects on central synapses after initial exposure remain poorly understood. We investigated the cellular and synaptic consequences of SC initiation in the prefrontal cortex of adolescent male mice. A single exposure to a prototypic SC reduced the expression of reelin, an extracellular matrix protein, via cannabinoid CB_1_ receptors in GABAergic neurons, impairing prefrontal synaptic function. Infusion of reelin restored normal plasticity. These findings provide new insights into the neural consequences of adolescent cannabinoid consumption and emphasize reelin’s key role in the cellular mechanisms associated with SC initiation during adolescence.

Drug initiation, including the first experimentation with a rewarding substance, sets the stage for subsequent behavioral patterns. Even a single encounter with a substance can induce enduring changes in the central nervous system that persist beyond the immediate presence of the drug (1, 2, 3, 4). This holds true during cannabis initiation, where the psychoactive properties of the plant trigger a cascade of biochemical, neuronal, and synaptic consequences. Novel psychoactive substances, a rising public health concern, emulate naturally existing molecules or conventional illicit psychoactive substances (5,6). Synthetic cannabinoids (SCs; also known as Spice) are intentionally designed to mimic the active components in natural cannabis (5,7). Recreational use of SCs has increased considerably in recent years, and the prevalence of SC use tends to be higher among young males (5,7, 8, 9).

Considering the acknowledged risks associated with SC use during adolescence (8,10) and the substantial prevalence of experimentation during this critical developmental period, it is crucial to address the lack of understanding regarding the effects of SCs initiation on central synapses. This knowledge void is especially critical in relation to the prefrontal cortex (PFC). The PFC is a brain region essential for decision making, impulse control, and social interaction, which undergoes significant development throughout adolescence and early adulthood in both humans and rodents, making it one of the last structures in the central nervous system to mature (11,12). This developmental period is characterized by profound changes in PFC structure and function, including the refinement of PFC connectivity and the maturation of cognitive, emotional, and social abilities in both species (13,14). Concurrently, adolescence is marked by increased exposure to new and diverse environmental factors, which extends the vulnerability of the PFC to various environmental insults, notably exogenous cannabinoids (10,15,16). Exposure to cannabinoids during adolescence causes lasting alterations in PFC-dependent behaviors, as well as abnormalities in prefrontal architecture and synaptic functions (17, 18, 19, 20, 21). The endocannabinoid (eCB) system, which includes G protein–coupled cannabinoid CB_1_ and CB_2_ receptors (CB1R and CB2R) and endogenous cannabinoid ligands, among other components, plays a significant role in these processes (22). The PFC exhibits a high density of CB1Rs (23,24), rendering it susceptible to eCB synaptic dysfunctions from SCs that result in various cognitive alterations (25). To understand the relationship between SC exposure during the critical period of adolescence, altered behavior, and abnormalities in the prefrontal circuitry, identifying the specific molecular mechanisms involved is necessary.

Reelin, a secreted glycoprotein of the extracellular matrix, plays a pivotal role in neuronal migration and layer formation during embryonic development (26). As a synaptic organizer, reelin is essential for the proper functioning of both postnatal and adult brain physiology. Reelin regulates the developmental trajectory of pyramidal PFC neurons and associated behaviors (27, 28, 29). During adolescence, reelin haploinsufficiency has adverse effects on synaptic transmission, excitatory-inhibitory balance, plasticity, and PFC-dependent behaviors (27,29). Furthermore, reelin is a critical molecular mediator of PFC dysfunction due to nutritional stress (15) and influences behavioral abnormalities associated with high Δ^9^-tetrahydrocannabinol (THC) use in adolescence (30). Considering the vulnerability of reelin to early-life environmental insults (15,31, 32, 33, 34), we hypothesized that it could serve as a link between initiation of SC use during adolescence and prefrontal dysfunctions. Because SCs consumption is more prevalent among young men (7,8), we focused our study on investigating the effects of SC exposure on reelin expression and PFC functioning in male adolescent mice.

## Methods and Materials

### Animals and Drug Treatments

C57BL/6J male mice (Janvier Labs) were received and left undisturbed for 7 days before drug administration. The reelin haploinsufficient heterozygous reeler mice (HRM) were obtained from HRM breeding pairs purchased from Jackson Laboratory (B6C3Fe a/a-Relnrl/J strain). Details are available in the Supplement.

Immunohistochemistry and image analysis is detailed in the Supplement.

### Electrophysiology

Coronal slices containing the prelimbic area of the medial PFC (later referred to as PFC) were prepared as previously described (28). More detailed information is available in the Supplement.

### Single-Molecule Fluorescent In Situ Hybridization

The molecular identity of *Reln*-expressing cells was determined using single-molecule fluorescent in situ hybridization (smFISH) (35), which is detailed in the Supplement. Multiple correspondence analysis followed by hierarchical clustering on principal component analysis was performed using the FactoMineR package in R (36).

Intra-PFC recombinant reelin infusion was performed as previously described (15) using stereotaxic coordinates based on Paxinos and Franklin’s mouse brain atlas (37). More details are available in the Supplement.

### Western Blots

Immunoblotting of reelin in whole PFC lysates was performed following previously published procedures (38).

Quantitative reverse transcriptase–polymerase chain reaction is detailed in the Supplement.

### Statistical Analysis

Statistical analysis was performed with GraphPad Prism version 10.3.1 (GraphPad Software) (see the Supplement).

## Results

### Effect of a Single In Vivo SC Exposure on Reelin’s Cellular Density in Specific PFC Layers

**Figure 1:**
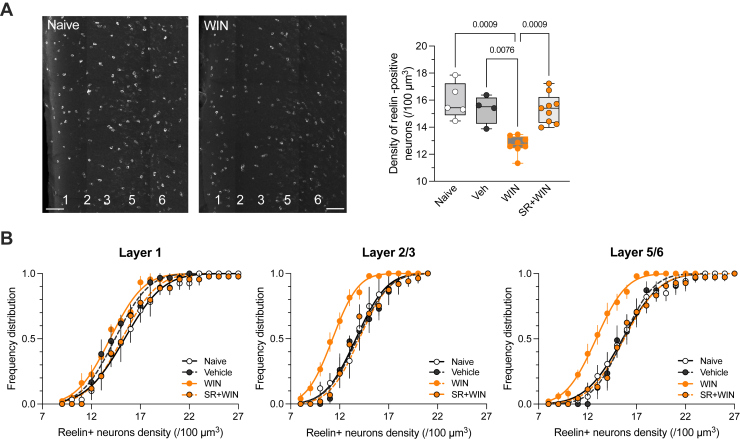
Single in vivo synthetic cannabinoid exposure affects the cellular density of reelin in specific PFC layers. **(A)** Confocal microscopy mosaic images of the prelimbic PFC area stained to visualize reelin in P40 naïve and WIN-treated mice (scale bars = 100 μm). Box and whiskers plot (box: median, 25th–75th percentiles; whiskers: minimum to maximum) showing the density of reelin-positive cells/volume as well as individual values obtained for each mouse. The median was 15.4 in naïve mice (*n* = 5 mice), 15.5 in vehicle-treated mice (*n* = 4 mice), 12.7 in WIN-treated mice (*n* = 8 mice), and 15.4 in SR+WIN-treated mice (*n* = 9 mice) (η^2^ = 0.603, *p* = .001, Kruskal-Wallis test). Only *p* values < .05 are indicated. **(B)** Cumulative distributions (means ± SEM) and fitting curves of the density of reelin-positive cells across the different PFC layers. The cumulative distributions reveal a specific effect of WIN in layers 2/3 and layers 5/6. The effect of WIN was blocked by the CB_1_ receptor antagonist SR. PFC, prefrontal cortex; SR, SR141716A; Veh, vehicle; WIN, WIN 55,212-2.

Cannabis and SCs acutely impair cognitive functions such as attention, memory, and decision making in humans and animals (10); however, research on persistent effects (i.e., one or more days after use) of the first exposure are limited (2,21,39,40). We hypothesized that cannabinoids may interact with the reelin system in the PFC. We chose to utilize the SC WIN 55,212-2 (WIN) instead of THC, the principle psychoactive component of cannabis, due to its increased potency for CB1R, as well as the public health concerns associated with SCs. Adolescent male mice were administered a single intraperitoneal injection of WIN or its vehicle, and the reelin expression pattern in the PFC was studied the following day by immunofluorescence. SC exposure led to an 18.2% ± 1.6% reduction in the density of reelin-positive cells throughout the entire PFC compared with naïve and vehicle-treated mice (Figure 1A). To investigate whether the observed effect was consistent across the PFC layers, we examined the cumulative distributions of reelin-positive cell densities in naïve mice, vehicle-treated mice, and WIN-treated mice. In layer 1, these distributions were superimposable, indicating no WIN effect in the most superficial layer of the PFC (Figure 1B). Conversely, in layers 2/3 and 5/6, the WIN-treated group distribution shifted to the left compared with naïve and vehicle-treated mice, suggesting decreased reelin-positive cell density in these layers due to WIN exposure (Figure 1B). Co-administration of the CB1R antagonist SR141716A with WIN prevented the total density reduction of reelin-positive cells (Figure 1A) and normalized the density distributions in layers 2/3 and 5/6, as evidenced by the overlap of SR+WIN, vehicle, and naïve cumulative probability curves (Figure 1B). Therefore, a single administration of an SC to a drug-naïve adolescent male mouse significantly disrupted reelin PFC expression in a layer-specific pattern through the activation of CB1Rs.

### RNAscope Profiling Reveals a Significant Prevalence of CB1R and Reelin Coexpression in PFC Neurons

**Figure 2:**
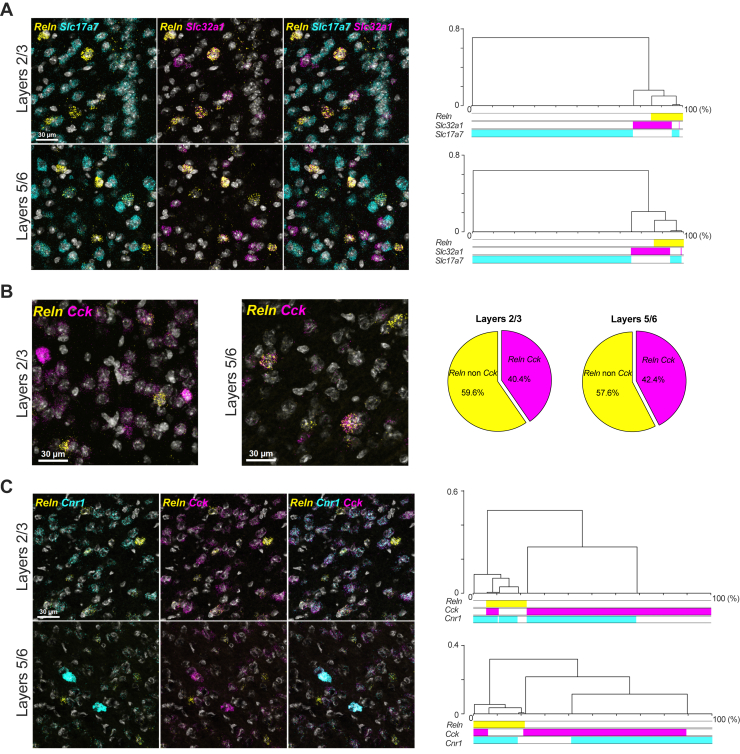
Profiling of reelin expression in the prefrontal cortex. Single-molecular fluorescent in situ hybridization of *Reln*, *Slc32a1*, *Slc17a1*, *Cck*, *and Cnr1* mRNA in the prelimbic area of the prefrontal cortex. Slides were counterstained with DAPI (white). **(A)** Dendrograms of hierarchical clustering applied to principal component analysis of mRNA distribution profiles in superficial (layers 2/3) and deep (layers 5/6) layers. The x-axis represents the cumulative distribution as percentage, and the y-axis quantifies the dissimilarity between neurons. The colored bars indicate the mRNA composition related to each cluster. Layers 2/3: *n* = 70 *Reln*-, *n* = 416 *Slc17a1*-, and *n* = 87 *Slc32a1*-positive neurons. Layers 5/6: *n* = 72 *Reln*-, *n* = 370 *Slc17a1*-, and *n* = 97 *Slc32a1*-positive neurons. **(B)** Pie graph depicting the proportion (%) of *Reln* mRNA-positive neurons coexpressing (pink) or not (yellow) *Cck* mRNA across the different layers. Layers 2/3: 45.8%, *n* = 118 *Reln*-positive neurons and layers 5/6: 51.6% *n* = 91 *Reln*-positive neurons. **(C)** Dendrograms of hierarchical clustering on principal component analysis of mRNA distribution analysis in superficial and deep layers. Layers 2/3: *n* = 46 *Reln*-, *n* = 233 *Cnr1*-, and *n* = 287 *Cck*-positive neurons and layers 5/6: *n* = 16 *Reln*-, *n* = 96 *Cnr1*-, and *n* = 101 *Cck*-positive neurons. mRNA, messenger RNA.

The layer-specific decrease in reelin levels induced by the SC prompted us to investigate the expression profile of reelin in the PFC, particularly its spatial and cellular association with the CB1R. SmFISH analysis was used to explore the specific RNA coexpression patterns of reelin (*Reln*) and various neuronal markers in the PFC of naïve male mice, covering layers 2 to 6 (Figure 2 and Figure S1). We found that *Reln*-positive neurons are molecularly heterogeneous, expressing GABAergic (*Slc32a1*) or glutamatergic (*Slc17a7*) markers (Figure 2). In terms of distribution, *Reln*-positive cells are primarily positive for *Slc32a1*, and their presence among GABAergic or glutamatergic populations is similar in both superficial layers (layers 2/3) and deep layers (layers 5/6) of the PFC (Figure 2A). *Reln* is coexpressed with the neuropeptide cholecystokinin (*Cck*), and the proportion of *Reln* neurons positive for *Cck* was similar across layers (Figure 2B). Considering the observed reduction in reelin expression upon CB1R activation (Figure 1), the significant proportion of *Reln*-positive neurons expressing *Cck* in the PFC (Figure 2B), and previous studies showing a strong coexpression of CCK with CB1R in various forebrain regions (41,42), we investigated the presence of CB1Rs (*Cnr1*) in *Reln*-positive neurons. The CB1R was expressed across all layers 2 to 6, and *Reln* demonstrated a high level of coexpression with the *Cnr1* in both superficial and deep layers (Figure 2C). Additionally, reelin was found to be expressed with neuropeptide Y (*NPY*) and somatostatin (*Sst*) (Figure S1).

These findings demonstrate a diverse array of *Reln*-positive neurons in the layers of the adolescent mouse PFC, exhibiting unique coexpression patterns with the *Cnr1* and/or *Cck*. Furthermore, our results revealed the presence of *Reln* in glutamatergic neurons within the PFC. Given the observed coexpression of the CB1R and reelin in prefrontal neurons, next we investigated how adolescent cannabinoid exposure interferes with reelin expression and function in the PFC.

### Cannabinoid-Induced Reduction in PFC Reelin Expression Is Selectively Mediated Through the Activation of CB1Rs on GABAergic Neurons

**Figure 3:**
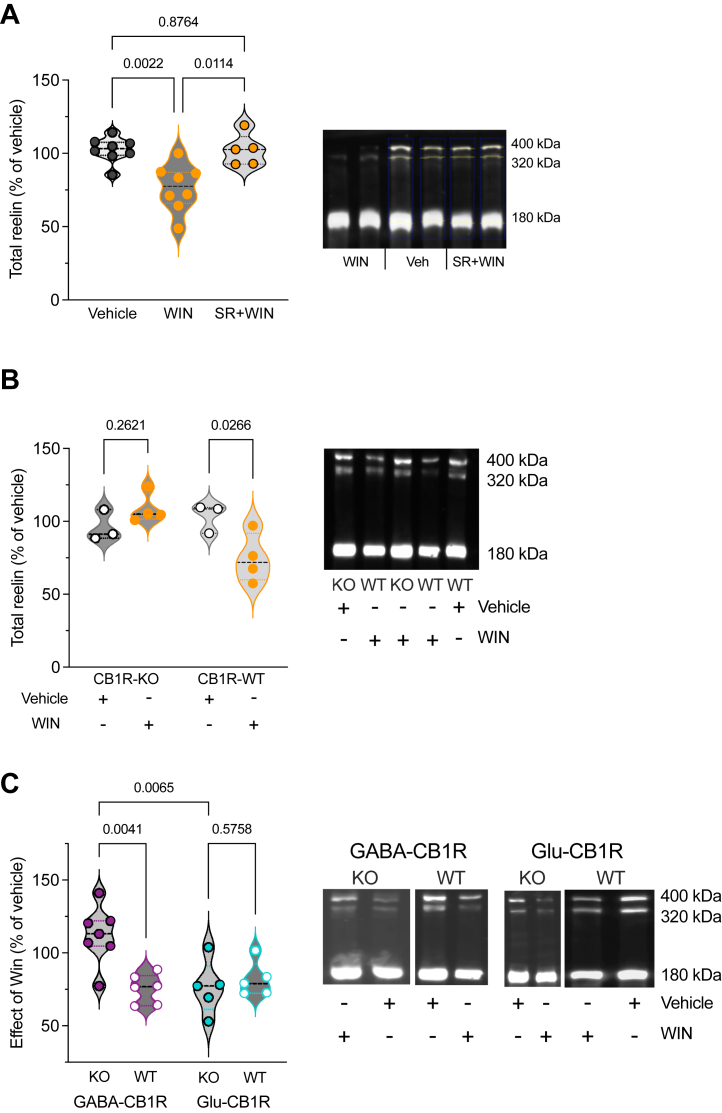
CB1R deletion in GABAergic neurons abolishes the reduction in prefrontal reelin induced by single in vivo synthetic cannabinoid exposure. **(A)** Violin plot showing the relative changes in reelin levels compared with the vehicle control group. Densitometric measurements of total reelin levels (sum of the 3 isoforms) expressed as the percentage of total reelin levels in vehicle-treated mice. The data show that WIN decreased reelin levels (WIN *n* = 8 mice) compared with vehicle-treated mice (*n* = 8 mice), and this effect was prevented by the antagonist SR (SR+WIN *n* = 5 mice) (η^2^ = 0.507, *p* = .001, Kruskal-Wallis test). Representative immunoblotting of PFC lysates extracted from vehicle-, WIN-, and SR+WIN-exposed mice and probed with the anti-reelin G10. Each lane is a different mouse. **(B)** Densitometric analysis of total reelin levels in WIN-injected CB1R-KO mice (*n* = 4 mice) and their wild-type littermates (CB1R-WT, *n* = 4 mice) compared with respective controls (vehicle-injected CB1R-KO, *n* = 3 mice and vehicle-injected CB1R-WT, *n* = 3 mice). WIN administration did not reduce reelin levels in CB1R-KO mice compared with WT littermates (η^2^ = 0.435, *p* = .0397, Kruskal-Wallis test). Representative immunoblotting of PFC lysates extracted from vehicle- or WIN-exposed CB1R-KO and -WT littermates, probed with the anti-reelin G10. Each lane is a different mouse. **(C)** Densitometric measurements of WIN effect on total reelin levels expressed as the percentage of total reelin levels measured in corresponding vehicle-treated control mice. The effect of WIN was selectively blocked in GABA-CB1R-KO (*n* = 7 mice) compared with Glu-CB1R-KO (*n* = 5 mice) and respective WT littermates (GABA-CB1R-WT, *n* = 6 mice and Glu-CB1R-WT, *n* = 5 mice) (η^2^ = 0.429, *p* = .0109, Kruskal-Wallis test). Representative immunoblotting of PFC lysates extracted from vehicle- or WIN-exposed GABA-CB1R-KO and Glu-CB1R-KO and WT littermates (GABA-CB1R-WT and Glu-CB1R-WT, respectively), probed with the anti-reelin G10. Each lane is a different mouse. CB1R, CB_1_ receptor; GABA, gamma-aminobutyric acid; KO, knockout; PFC, prefrontal cortex; SR, SR141716A; WIN, WIN 55,212-2; WT, wild-type.

We found that the reduction in PFC reelin-positive cell density following WIN exposure correlated with alterations in reelin cellular protein levels. Initially, Western blot analysis of whole PFC lysates using the anti-reelin G10 antibody revealed that WIN treatment decreased reelin levels compared with mice treated with vehicle alone. Importantly, this effect was effectively blocked by the CB1R antagonist SR141716A (Figure 3A). Furthermore, densitometric analysis of total reelin levels in mice exposed to the SC revealed that the reduction of reelin levels induced by WIN was not present in global CB1R knockout mice (CB1R-KO) in comparison to their wild-type littermates (Figure 3B). These findings, which complement the immunostaining observations (Figure 1), demonstrate that in the PFC, the SC acted on CB1Rs to decrease reelin protein levels.

Next, we aimed to obtain genetic confirmation substantiated by our smFISH analysis suggesting that most neurons coexpressing reelin and CB1Rs in the PFC are of GABAergic nature (Figure 2). To specify the cell types of CB1R-expressing neurons responsible for the reduction in reelin expression, we utilized conditional knockouts targeting either CB1Rs in forebrain GABAergic (i.e., Dlx5/6-CB1R-KO mice, named GABA-CB1R-KO) or cortical glutamatergic (i.e., Nex-CB1R-KO mice, named Glu-CB1R-KO) neurons (43, 44, 45). The data showed that the effects of WIN on the day after were preserved in Glu-CB1R-KO mice, whereas these effects were abolished in GABA-CB1R-KO mice (Figure 3C). These findings strongly suggest that the reduction of prefrontal reelin expression by cannabinoids relies on the activation of the CB1R present on GABAergic neurons.

### Single SC Exposure Modifies the Proteolytic Fragmentation of Reelin Rather Than Its Gene Expression

**Figure 4:**
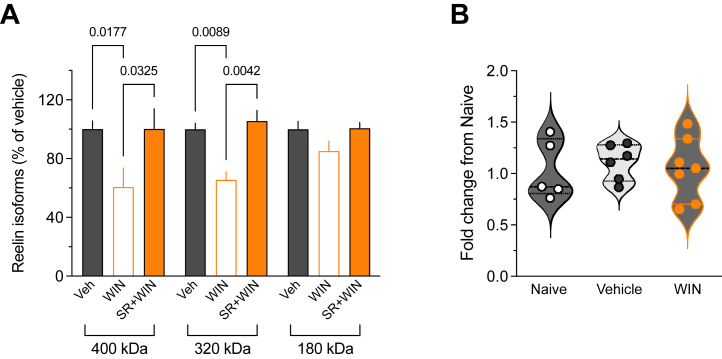
Synthetic cannabinoid exposure modifies the proteolytic processing of reelin. **(A)** Densitometric analysis of full-length reelin (400 kDa) and the 2 cleaved products (320 and 180 kDa) detected by the G10 antibody in WIN-treated (*n* = 8 mice), SR+WIN-treated (*n* = 5 mice), and vehicle-treated (*n* = 8 mice). Histogram showing means ± SEM of individual values expressed as the percentage of the corresponding reelin isoform detected in the vehicle-treated group (η^2^ = 0.423, *p* = .0065, Kruskal-Wallis test). Only *p* values < .05 are indicated. **(B)** Quantitative polymerase chain reaction analysis of *Reln* messenger RNA in prefrontal cortex tissue collected from naïve, vehicle-, and WIN-treated mice (*n* = 5, 6, and 7 mice, respectively). *Reln* messenger RNA expression was not different among these 3 groups (*p* = .8062, Kruskal-Wallis test). SR, SR141716A; Veh, vehicle; WIN, WIN 55,212-2.

In the developing and adult brains, the full-length reelin protein (with relative molecular masses of 388 kDa and 450 kDa when glycosylated) (46) undergoes secretion and enzymatic cleavage at 2 primary sites, producing 5 fragments (47,48). In addition to the full-length reelin, the G10 antibody can detect 2 N-terminal region fragments (38,49,50). Analysis of the forms recognized by the G10 antibody revealed reduced amounts of full-length protein and the 320-kDa cleavage product in the WIN-exposed group (Figure 4A), suggesting diminished secretion and impacted proteolytic cleavage following CB1R activation. To further elucidate the mechanism that underlies reelin downregulation in SC-exposed mice, we performed quantitative polymerase chain reaction on the PFC from naïve, vehicle-, and WIN-exposed mice. Despite the delta delta CT variance, the study is adequately powered (1 − beta = 0.97) to detect a major effect in transcripts levels. No differences in *Reln* messenger RNA (mRNA) levels were observed across groups (Figure 4B), suggesting that adolescent cannabinoid initiation did not significantly influence reelin transcription. Taken together, the data support the idea that SC reduces reelin levels by disrupting its proteolytic cleavage rather than by altering its transcription.

### Single In Vivo SC Exposure Durably Reduces PFC Long-Term Potentiation

Altered PFC long-term synaptic plasticity is commonly observed in various models of neurodevelopmental diseases, perinatal insults, and adolescent insults (15,16,21). In a previous study using reelin haploinsufficient mice, we demonstrated the abolishment of theta burst stimulation (TBS)–induced long-term potentiation (TBS-LTP) in the PFC (28). Building on this, we hypothesized that the reduction of reelin levels, triggered by SC and mediated through the CB1R, impacts TBS-LTP.

**Figure 5:**
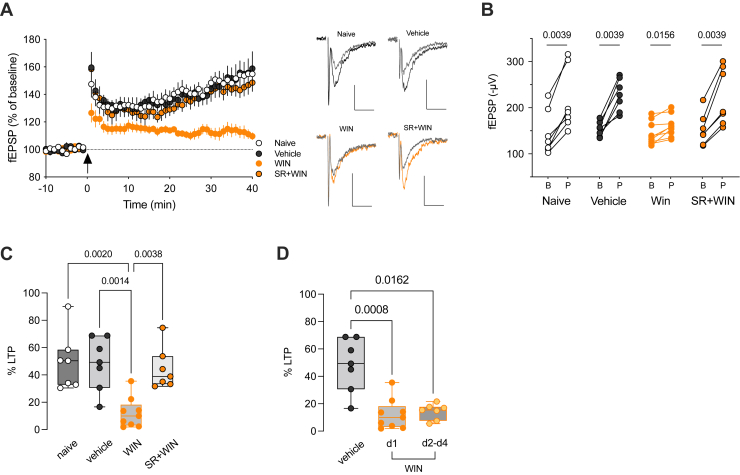
Single in vivo synthetic cannabinoid exposure durably impairs adolescent prefrontal LTP. **(A)** Grouped time courses (average ± SEM) of fEPSP responses expressed as the percentage of baseline before and after TBS (indicated by arrow). TBS induced LTP with similar time courses in naïve (*n* = 7 mice), vehicle- (*n* = 7 mice), and SR+WIN-treated mice (*n* = 7 mice). TBS-LTP was reduced in WIN-exposed mice (*n* = 9 mice). Representative traces averaged from 10 fEPSP responses before (gray) and 40 minutes after plasticity induction. Stimulation artifacts were blanked. Calibration: 100 μV, 10 ms. **(B)** fEPSP amplitudes of individual experiments during baseline and 30 to 40 minutes post TBS. Wilcoxon tests. **(C)** Box (median, 25th–75th percentiles) and whiskers (minimum to maximum) plot with individual values showing the percentage of potentiation measured 30 to 40 minutes after TBS (η^2^ = 0.463, *p* = .0018, Kruskal-Wallis test). Only *p* values < .05 are indicated. **(D)** Box plot showing that the reduction of TBS-LTP by WIN lasted up to the fourth day post intraperitoneal injection. The percentage of TBS-LTP decreased to 14.22% ± 2.16% (*n* = 7 mice) between 2 and 4 days after WIN injection. This level is comparable to the mean ± SEM observed 17 ± 1 hours after WIN exposure (12.2% ± 3.7%, *n* = 9 mice; day 1). Circles indicate individual values obtained for each mouse (η^2^ = 0.491, *p* = .0027, Kruskal-Wallis test). Only *p* values < .05 are displayed. B, baseline; d, day; fEPSP, field excitatory postsynaptic potential; LTP, long-term potentiation; P, post TBS; SR, SR141716A; TBS, theta burst stimulation; WIN, WIN 55,212-2.

We observed a significant reduction in the magnitude of TBS-LTP at deep PFC excitatory synapses following a single injection of WIN, compared with naïve and vehicle-treated mice, the day after SC administration (Figure 5A–C). Notably, the LTP reduction was absent when the SC was administered together with a CB1R antagonist (Figure 5A–C), suggesting that CB1R activation plays a crucial role in the detrimental effects of WIN on prefrontal LTP. Notably, the decrease in TBS-LTP caused by a single SC exposure remained evident even on the fourth day following SC administration, indicating that the impact of in vivo SC on synaptic plasticity persisted long after the first encounter with the drug (Figure 5D). Variations in the excitability of layer 3/5 excitatory synapses cannot account for this effect because the input-output curves were found to be identical across all experimental groups (Figure S2).

In the PFC, TBS-LTP relies on NMDA receptor (NMDAR) activation (29). To explore whether the effects of WIN on deep layer excitatory synapses were associated with changes in NMDARs, we assessed the transcriptional levels of NMDAR subunits using quantitative polymerase chain reaction analysis. The results revealed no significant differences in mRNA levels of NMDAR subunits in the PFC of WIN-treated mice compared with the control groups (Figure S3), indicating that the reduction in TBS-LTP was not a result of altered transcription of NMDAR subunits.

### Reelin Deficiency Underlies SC-Induced Impairment of Synaptic Plasticity

Our results thus far indicate that a single SC administration to drug-naïve adolescent male mice diminishes the density of reelin-positive cells in specific cortical layers through CB1R-mediated modification of reelin secretion and/or proteolytic cleavage. Furthermore, this SC exposure persistently impairs LTP.

To establish a functional relationship between CB1R-induced reelin reduction and LTP impairment, 2 specific predictions need to be satisfied: 1) LTP is impaired in another model of reelin reduction, and 2) intra-PFC supplementation of reelin will protect from the loss of LTP induced by SC exposure.

**Figure 6:**
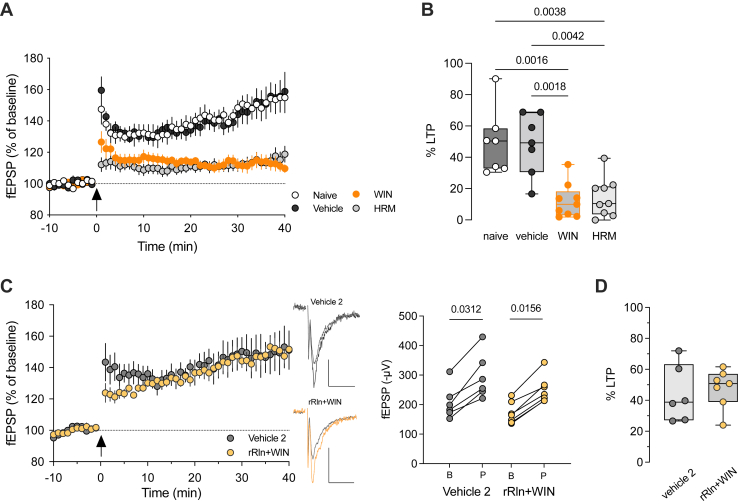
Prefrontal cortex administration of recombinant reelin prevents LTP impairment induced by synthetic cannabinoid exposure. **(A)** Average (±SEM) time courses of fEPSPs normalized to respective baselines in naïve-, vehicle-, WIN-treated, and adolescent HRM male mice. **(B)** Box (median, 25th–75th percentiles) and whiskers (minimum to maxixum) plot with individual values (mice) for each experimental group showing the percentage of potentiation obtained 30 to 40 minutes after TBS (η^2^ = 0.542, *p* = .0004, Kruskal-Wallis test). **(C)** Average (±SEM) time courses of normalized fEPSP responses showing similar TBS-LTP in rRln+WIN-treated mice (*n* = 7 slices/5 mice) compared with vehicle 2-treated mice (*n* = 6 slices/3 mice). Representative traces averaged from 10 fEPSP responses before (gray) and 40 minutes after TBS. Stimulation artifacts were blanked. Calibration: 100 μV, 10 ms. Right: fEPSP amplitudes of individual experiments before and 30 to 40 minutes after TBS in rRln+WIN- or vehicle 2-treated mice. Wilcoxon tests. **(D)** Box and whiskers plot (box: median, 25th–75th percentiles; whiskers: minimum to maximum) with individual values showing the percentage of potentiation measured 30 to 40 minutes after TBS. Mann-Whitney test. Only *p* values < .05 are displayed. B, baseline; fEPSP, field excitatory postsynaptic potential; HRM, heterozygous reeler mouse; LTP, long-term potentiation; P, post TBS; rRln, recombinant reelin; TBS, theta burst stimulation; WIN, WIN 55,212-2.

To address the first prediction, we investigated TBS-induced plasticity in a mutant mouse haploinsufficient for reelin (HRM) (28), aiming to explore the connection between reelin deficiency and decreased TBS-LTP after SC administration. We found that the time course and magnitude of TBS-LTP in adolescent HRM male mice closely resembled those observed in animals exposed to WIN (percentage of TBS-LTP: 12.2% ± 3.7%, *n* = 9 WIN-treated vs. 14.4% ± 4.1%, *n* = 9 HRM) (Figure 6A, B). Thus, a genetic reduction of endogenous reelin mimics the harmful effects of SC on PFC plasticity. Therefore, the alteration of TBS-induced plasticity following in vivo exposure to WIN is consistent with a decrease in endogenous reelin levels triggered by WIN exposure.

To validate that the decline in TBS-LTP after exposure to WIN was caused by reduced reelin expression, recombinant reelin (rRln) was bilaterally injected into the PFC, as we had done previously (15). Adolescent mice received WIN injections 2 days after rRln administration, and TBS-LTP was evaluated 17 ± 1 hours after WIN exposure. Administering rRln into the PFC completely prevented the WIN-induced decrease in TBS-LTP, restoring LTP magnitude to normal levels (Figure 6C, D). Importantly, this effect was not attributed to the influence of rRln on the excitability of layer 3/5 excitatory synapses because input-output curves were identical among all groups (Figure S4). These results further support the observation that reelin downregulation functionally contributes to the synaptic impairments induced by SC exposure.

## Discussion

Although SCs and cannabis have become popular among adolescents for recreational use, the long-term effects of the initial exposure to these substances remain poorly understood. Despite the increasing prevalence of SCs and their growing use especially among young men, the consequences of first-time exposure have been largely overlooked in research.

In this study, we investigated the consequences of an initial exposure to a prototypic SC, WIN 55,212-2, during adolescence using drug-naïve male mice as a model. Our findings revealed a decrease in reelin expression in specific layers of the prelimbic area of the medial PFC and a disruption in reelin protein fragmentation with no significant change in its mRNA expression. RNAscope profiling revealed that *Reln* transcripts were enriched in GABAergic cells expressing CB1Rs and CCK and to a lesser extent in glutamatergic pyramidal neurons. Pharmacological and genetic approaches revealed that the SC-induced decrease in reelin was due to the activation of CB1Rs expressed on GABAergic PFC neurons. Additional electrophysiological assessments of glutamatergic synaptic functions in the PFC demonstrated that a single exposure to SC caused a lasting decrease in PFC LTP, similar to observations in reelin haploinsufficient mice. We also demonstrated that intra-PFC administration of reelin is sufficient to prevent SC-induced LTP deficits. Our results strongly suggest that reduced reelin levels in the PFC are the primary cellular mechanism that underlies the enduring effects of initial SC exposure on adolescent PFC synaptic plasticity.

### Heterogeneity of Prefrontal Reelin-Expressing Cells

Immunolabeling revealed the presence of reelin-expressing cells dispersed across all layers of the adolescent PFC (Figure 1), mirroring the widespread distribution that has been observed in the frontoparietal (51) and iso/neo cortices of the adult rodent brain (52,53).

Using smFISH, we phenotyped cells synthetizing reelin in the mouse PFC (Figure 2). This approach showed that in the prefrontal region, reelin is produced both by GABAergic and glutamatergic neurons, identified by *Slc32a1* and *Slc17a1* markers, respectively.

The expression of *Reln* mRNA was predominantly detected in interneurons identified by *Slc32a1* in both the superficial layers 2/3 and deep layers 5/6 (∼67.2% and ∼57.3%, respectively). *Reln* mRNA was also observed, although to a lesser extent, in excitatory glutamatergic neurons found both in superficial (∼23.9%) and deep (∼38.7%) layers. In most of the postnatal brain, reelin is expressed by GABAergic interneurons (51, 52, 53). Our findings extend previous research showing that reelin is also expressed by non-GABAergic pyramidal neurons in the hippocampal formation of nonhuman primates (54) and in layer 2 of the entorhinal cortex and layer 5 of the isocortex in rats (51,53).

Furthermore, the molecular phenotyping revealed a broad expression of *Cck* mRNA throughout the prelimbic PFC, characterized by a strong colocalization of *Reln* transcripts with *Cck*. This pattern is distinctive for the PFC because reelin and CCK are rarely coexpressed in regions such as the hippocampus and frontoparietal and barrel cortices (51,52,55). Consistent with previous studies that have shown high expression of CB1Rs in GABA interneurons that contain CCK (23,56,57), we found that most *Cnr1*-expressing neurons were also *Cck*-expressing interneurons (∼79.2% superficial layers and ∼70.1% in deep layers). Furthermore, consistent with this anatomical localization, we observed that *Cnr1* and *Reln* were highly coexpressed in both superficial and deep layers (∼75.0% and ∼86.3%, respectively). While previous studies have reported expression of CB1Rs in reelin-expressing Cajal-Retzius and migratory cells (58,59) during embryonic stages in the mouse telencephalon, our findings represent the first documentation of an enrichment of CB1Rs in postnatal prefrontal reelin–expressing neurons.

### CB1Rs on GABAergic Neurons Regulate Reelin Expression

This study provides the first evidence that SC activation of CB1Rs downregulates reelin expression in the PFC in a layer-specific manner (Figure 1). We previously showed that in the PFC, CB1Rs are selectively expressed in layers 2/3 and 5/6 (24), consistent with the lack of effect of SC on reelin expression in layer 1.

Given the expression of CB1Rs across excitatory and inhibitory neurons (23,44,60), we sought to determine the relative contribution of these two cell types in the SC-induced decrease in reelin expression. The reduction in reelin levels induced by SC was absent when CB1R was selectively ablated from GABAergic neurons. However, this reduction was preserved in mice with a selective deletion of CB1Rs from principal excitatory neurons (Figure 3), indicating that the activation of CB1Rs on GABAergic neurons is responsible for the SC-induced changes in reelin levels. These findings were substantiated by smFISH analysis, which revealed the coexpression of *Cnr1* and *Reln* in *Cck* neurons. Given the widespread expression of CCK in both interneurons and pyramidal neurons (61), additional investigations will confirm whether reelin expression is specifically and/or selectively regulated by the CB1Rs expressed by CCK interneurons. Consistent with this notion and our findings, recent research has highlighted the role of CB1R located on CCK-expressing interneurons in the anterior cingulate cortex in social impairment in male mice (62).

### Consequences of SC Exposure on Network Plasticity

Deficits in synaptic plasticity following acute cannabinoid exposure, whether THC or WIN, depend on brain regions, age, and the type of plasticity involved. A single exposure to THC ablated eCB-mediated synaptic plasticity in the adult mouse nucleus accumbens and hippocampus 15 to 20 hours after exposure (2). However, this impairment was not observed for hippocampal CA1 LTP 24 hours after THC exposure in juvenile rats (39) or for eCB long-term depression in ventral tegmental area of juvenile-adolescent mice 24 hours post-THC injection (4). In adult rats, a single acute injection of WIN, 24 hours prior, impaired LTP in the ventral subiculum-accumbens pathway (40) and ablated the Schaffer collateral-CA1 LTP 30 minutes after injection in late adolescent rats (63). A single WIN exposure selectively ablated NMDAR LTP in the PFC of adult male rats 24 hours postexposure, but not in adolescent rats, whereas eCB-mediated long-term depression was unaffected in males of both ages (21).

Our data also demonstrate that a single in vivo pharmacological intervention can mimic the consequences of gene haploinsufficiency on prefrontal network plasticity, both in magnitude and duration. The approximately 20% reduction in the density of reelin-positive cells following exposure to SC was sufficient to reduce NMDAR-dependent long-term excitatory synaptic plasticity to levels observed in reelin haploinsufficient mice, an effect that lasted for several days. It is noteworthy that synaptic changes in PFC circuits can persist long after clearance of the drug (64) and thus cannot be explained by ongoing occupation of CB1Rs by WIN 17 ± 1 hours post intraperitoneal administration.

### Potential Mechanisms

To date, no functional connection between reelin and CB1Rs has been described aside from their coexpression in some cells at the embryonic stage (58,59). Our results provide the first documentation of such a functional connection. Although we have evidence regarding the mechanisms involved, additional experiments are necessary to fully elucidate them. Our data show that the activation of CB1Rs on GABAergic neurons decreases reelin expression, subsequently regulating the activity and function of neighboring glutamatergic synapses.

After its secretion, reelin is processed at 2 major sites to produce 5 fragments, of which only 2 fragments carrying the N-terminal epitope, N-R6 and N-R2, are detected by the G10 antibody (65). Following SC exposure, we observed a reduction in both levels of full-length reelin and the 320-kDa fragment (N-R6) (Figure 4). This reduction could be attributed to either decreased secretion of the full-length protein or increased N-terminal processing rather than diminished translation because *Reln* mRNA levels were comparable between control and SC-exposed groups. The fact that the levels of the 180-kDa fragment (N-R2) remained unchanged after SC exposure supports the notion of decreased secretion combined with increased N-terminal processing in the presence of SC. Previous studies have shown that reelin fragments carry distinct physiological functions during brain development and postnatal stages, and they also differ functionally from full-length reelin [reviewed in (26,66)]. The central repeat fragment is required for the functions of reelin during cortical development (65) and rescues behavioral deficits observed in HRM mice during adulthood (67). Given these facts and the observation that the levels of the N-R2 fragment do not change after SC exposure, we can hypothesize that full-length reelin and N-R6 (both containing the central repeat fragment), but not N-R2, are required for the adequate expression of synaptic plasticity at prefrontal glutamatergic synapses. Unlike full-length reelin, which is a large protein that dimerizes after secretion (68) and is thus expected to have reduced diffusion and act locally in proximity of the secreting cells (69), reelin fragments are anticipated to diffuse over larger distances and act more distantly (47,69). Reelin function is regulated by its proteolysis (70), and the N-terminal processing of reelin regulates the duration of downstream signaling and its distal localization (69). It is possible that in the absence of SC exposure, regulation of N-terminal cleavage may be important for controlling the diffusion of N-R6 from the site of reelin secretion, allowing it to reach and activate signaling at distant glutamatergic synapses (47,71) and facilitate TBS-LTP.

Previous work from our group has demonstrated that reelin assumes key roles in regulating excitatory and inhibitory neurotransmission, synaptic plasticity, and fear memories. Normal levels of reelin are necessary for the correct maturation of NMDARs and expression of NMDAR-dependent LTP in the PFC (27,28,38) and hippocampus (72). Our current results show that synaptic function is restored by reelin supplementation before SC exposure (Figure 6), suggesting that SC exposure impairs reelin signaling at prefrontal excitatory synapses. This impairment may occur through mechanisms previously reported by our group, which involve reduced synaptic mobility of surface NR2B-containing NMDARs following the blockade of reelin function in vitro (50) or changes in the synaptic content of glutamatergic ionotropic receptors leading to a reduction of the AMPA/NMDA ratio in HRM mice (29).

### Future Directions

While several studies (2,4,39,40,63), including the current work, have focused solely on males, sex-specific consequences of cannabinoid exposure have been reported. For example, Borsoi *et al.* (21) demonstrated that prefrontal eCB-mediated synaptic plasticity was specifically occluded in females, whereas prefrontal LTP was selectively impaired in males following SC exposure. In humans, sex differences in the clinical outcomes of SC use have been observed (9), and in murine models, sex differences in SC self-administration behavior have been reported (73). Furthermore, Iemolo *et al.* (30) showed sex differences in cognitive and emotional behavior in HRM mice chronically exposed to THC, suggesting that sex differences may also exist in reelin depletion following SC exposure. In the current study, only male mice were tested, which is a limitation but also an opportunity for future research to explore sex-specific effects of SC exposure, as highlighted by the aforementioned studies.

These studies (2,4,21,39,40,63) have also described age-related differences in the effects of cannabinoid exposure on synaptic plasticity. The current work focused on adolescence and did not assess adult mice, leaving it unclear whether the observed effects of SC exposure on reelin are specific to adolescence or extend into adulthood. Future research should investigate the consequences of SC exposure in adulthood in both male and female mice to address potential sex- and age-related differences.

Many SCs are available, including AM678 (JWH-018), a cannabinergic aminoalkylindole frequently found in recreational products like Spice (74). Because both JWH-018 and WIN 55,212-2 interact with CB1Rs, future experiments that compare their effects on synaptic plasticity and reelin expression could provide valuable insights into whether their mechanisms of action are similar and potentially inform translational applications.

This study, which is the first to investigate the relationship between reelin dysregulation and SC exposure, suggests a potential mechanistic link between SCs and schizophrenia-like symptoms. Reelin, a protein crucial for brain development, is often found to be reduced in various brain regions of individuals with psychiatric disorders like schizophrenia, bipolar disorder, and major depression (75,76). This reduction in reelin expression, coupled with the known association between adolescent SC use and increased risks for mental health problems, including schizophrenia-like episodes (77, 78, 79), supports the potential link suggested by this study. Further research is needed to examine the intersection of synthetic and natural cannabinoids, adolescent vulnerability, and disorders associated with both low reelin expression and cannabinoid use. By comparing the effects of synthetic and natural cannabinoids, we can gain a deeper understanding of the pathophysiology of these conditions.

### Conclusions

Overall, our findings highlight the detrimental effects of SC initiation and establish a causal link between CB1R activation and prefrontal reelin deficits. They emphasize the cellular mechanisms and importance of reelin in the enduring effects of initial SC exposure on synaptic plasticity in the adolescent PFC. In conjunction with our previous study (15), these results suggest that reelin is a sensitive target of various early-life environmental insults, including nutritional stress and drug exposure. Thus, reelin may serve as a neurobiological hub that underlies the development of psychiatric diseases.

## Supplementary Material

Supplemental Methods and Materials and Figures S1–S4

Key Resources Table
