# Supplementary material for: Reelin Deficiency and Synaptic Impairment in the Adolescent Prefrontal Cortex Following Initial Synthetic Cannabinoid Exposure
Source: Biol Psychiatry Glob Open Sci. 2024 Nov 28;5(2):100426. doi: 10.1016/j.bpsgos.2024.100426 (PMC11804564; doi:10.1016/j.bpsgos.2024.100426)
Supplement: Supplemental Methods and Materials and Figures S1–S4 [file mmc1.pdf]

SUPPLEMENTARY INFORMATION

Reelin Deficiency and Synaptic Impairment in Adolescent Prefrontal Cortex Following Initial Synthetic Cannabinoid Exposure

Silva-Hurtado *et al.*

Contents :

Supplemental Methods and Materials

Supplementary Figures 1-4

Supplemental Methods and Materials.

Animals and drug treatments. Drugs were injected intraperitoneally between postnatal days 34 and 40. Injections were administered at the same time for all animals and aligned with the onset of the dark cycle. Mice were sacrificed 17 ± 1 hours after drug administration. Animals received a single intraperitoneal injection of WIN 55,212-2 (2 mg/kg) alone or with SR141716A (8 mg/kg). WIN 55,212-2 or SR141716A were suspended in 1:2:37 dimethyl sulfoxide, cremophor, and NaCl 0.9% (B. Braun), and injected at 5 mL/kg. Control mice received vehicle (1:2:37 dimethyl sulfoxide, cremophor, and NaCl 0.9%). Experimenters were blind to treatments. Offsprings from the HRM breeding were genotyped by PCR as previously described (1), weaned at 21 days, and then socially housed in same-sex groups. All mice were housed in standard 12h light–dark cycle and supplied food pellets and water *ad libitum*. Animals were treated in compliance with the European Communities Council Directive (86/609/EEC) and procedures carried out in accordance with protocols approved by The French Ethical committee (Authorization APAFIS#3279-2015121715284829 v5).

Immunohistochemistry. Animals were deeply anesthetized with Pentobarbital (90 mg/kg; Exagon Med'Vet) and perfused transcardially with cold phosphate-buffered saline solution (PBS; Gibco Life Technologies) followed with Antigenfix (DiaPath) a phosphate-buffered paraformaldehyde solution. The dissected brains were postfixed overnight at 4°C in the same fixative. Brains were then sectioned using a vibratome (VT 1200 s, Leica) into 60 µm-thick coronal slices. To obtain a whole rostro-caudal representation of the PFC of each mouse, coronal sections were collected from Bregma 2.58 mm to 1.94 mm. Free-floating sections were first rinsed three times for 10 min in PBS and then incubated in blocking solution with 0.1 M PBS containing 0.3% Triton X100 (Sigma) and 0.2% Bovine Serum Albumin (BSA; Sigma) twice for 1h at room temperature (RT). Slices were incubated overnight at RT with the mouse G10 anti-Reelin primary antibody (1:3000, MAB5364; Millipore) diluted in the blocking solution. After three blocking solution washes (10 min each), section was incubated at RT for 75 min with the secondary antibody goat anti-mouse Alexa 568 (Invitrogen ThermoFisher Scientific) diluted 1:500 in the blocking solution. After three rinses of 10 min in 0.1 M PBS, sections were stained with Hoechst (Invitrogen ThermoFisher Scientific) diluted 1:1000 in 0.1 M PBS for 12 min, washed again three times for 10 min in 0.1 M PBS and cover slipped with Aqua-Poly/Mount (Polysciences). The specificity of the G10 antibody was tested on sections obtained from homozygous reeler mice lacking Reelin expression. Additional negative controls were performed by omitting the G10 primary antibody (not shown).

Image analysis. Confocal images were acquired with a Zeiss LSM-800 system equipped with emission spectral detection and a tunable laser providing excitation range from 470 to 670 nm.

3x3 tiles stacks of optical sections containing the prelimbic area of the medial PFC (later referred to as ‘PFC’) were collected with a Plan Apochromat 20x.0.8 air objective for 3D reconstructions. Laser power and photomultiplier gain were adjusted to obtain a few pixels with maximum intensity on the somata showing the highest labelling intensity. To obtain a Z-representation across layers 1 to 6 within each brain section, scanning was performed using tiles representing a total of 894.4 µm x 894.4 µm surface/image size and a Z-stack selection covering a depth of 17.5 to 19.8 µm. The three-dimensional reconstruction and blind semiautomated analysis were performed with Fiji (2).

Electrophysiology. Briefly, mice were anesthetized with isoflurane and 300 µm-thick coronal slices were prepared in a sucrose-based solution at 4°C using an Integraslice vibratome (Campden Instruments). Slices were stored for 30 min at 32°C in artificial cerebrospinal fluid (ACSF) containing (in mM): NaCl (130), KCl (2.5), $MgCl_2$ (2.4), $CaCl_2$ (1.2), $NaHCO_3$ (23), NaH_2PO_4 (1.2) and Glucose (11), equilibrated with 95% O_2 / 5% CO_2. Slices were then stored at room temperature until recording. All experiments were conducted at 30-32°C in ACSF. For recording, slices were superfused at 2 ml per min with ACSF containing Picrotoxin (100µM; Sigma) or SR95531 (Gabazine, 5µM; Tocris) to block $GABA_A$ receptors. The prelimbic area of the PFC was visualized using an infrared illuminated upright microscope (Olympus BX51WI, France) and extracellular field excitatory postsynaptic potentials (fEPSPs) recordings carried out as previously described (1,3-6). fEPSPs were recorded in the PFC layer 5 with an ACSF-filled electrode and evoked in layer 3 at 0.1 Hz with a stimulating glass electrode filled with ACSF. The polarity of the stimulation was checked and adjusted for each experiment. LTP was induced using a theta-burst stimulation protocol consisting of five trains of burst with four pulses at 100 Hz, at 200 ms interval, repeated four times at intervals of 10 s (1,3). The glutamatergic nature of fEPSPs was confirmed at the end of each experiment by perfusing the non- N-methyl-D-aspartate (NMDA) ionotropic glutamate receptor antagonist 6-cyano-7-nitroquinoxaline-2,3-dione (CNQX) (20 µM; NIH), which specifically blocked the synaptic component without altering the non-synaptic component (not shown). Signals were collected using an Axopatch-200B amplifier (Axon Instruments, Molecular Devices, Sunnyvale, USA), filtered at 2 kHz, digitized at 10 kHz, acquired with Clampex 10.7 acquisition Software via a Digidata 1440A (Axon Instruments) and analyzed using Axograph 1.7.6.

Single molecule fluorescent *in situ* hybridization. Brains from C57BL/6J male mice were rapidly extracted and flash-frozen on aluminium foil on dry ice and stored at -80°C. Brains were cryostat sectioned at 16 µm and mounted directly onto Superfrost Plus slides (Fisherbrand). Coronal sections were collected from Bregma 2.10 mm to 1.94 mm. The following probes - *Reln* (ACDBio; Mm-reln-C3, Cat# 405981-C3), *Npy* (ACDBio; Mm-npy-C2, Cat# 313321-C2),

Calb2 (ACDBio; Mm-calb2, Cat# 313641), *Pvalb* (ACDBio; Mm-pvalb, Cat# 421931), *Sst* (ACDBio; Mm-sst-C2, Cat# 404631-C2), *Slc17a7* (ACDBio; Mm-slc17a7, Cat# 481851) and *Slc32a1* (ACDBio; Mm-slc32a1-C2, Cat# 319191-C2), *Cnr1* (ACDBio; Mm-cnr1, Cat# 420721), *Cck* (ACDBio; Mm-cck-C2, Cat# 402271-C2) - were used with the RNAscope Fluorescent Multiplex Kit (ACDBio; Cat# 320850) as previously described (7). Slices were counterstained for the nuclear marker with DAPI using ProLong Diamond Antifade mounting medium (Thermo Fisher scientific P36961). Confocal microscopy and image analyses were carried out at the Montpellier RIO imaging facility. Single confocal sections of double- and triple-labeled images from superficial and deep layers of the prelimbic area of the medial PFC were captured using sequential laser scanning confocal microscopy under a 40X objective (Leica SP8). Images were exported and counting was performed manually with Fiji (2). Quantification was performed out of 1 to 3 sections per mouse, from 3 mice per probe combination, to obtain the number of single, double and triple labelled neurons. Cells expressing at least one mRNA were assigned binary qualitative variables indicating the presence or absence of each mRNA.

Intra-PFC recombinant Reelin infusion. Microinjection needles (32 Ga) were connected to a 10 µl Hamilton syringe placed onto a microsyringe pump controller (Scientific Legato 130). The syringe was filled with recombinant mouse Reelin protein suspended in Phosphate Buffered Saline (rRln) (0.1 µg/µL; 0.75 µL; 3820-MR-025, R&D Systems) or vehicle 2 (Phosphate Buffered Saline). Mice were anaesthetized using 2-3% isoflurane in oxygen. Carprofen was administered prior to surgery as an analgesic and anti-inflammatory (Rimadyl, 5 mg/kg). Lidocaine was administered by subcutaneous injection for local anesthesia in the incision site. Mice were placed in a stereotaxic frame and bilaterally injected into the mPFC with rRln or vehicle (0.75 µL/side with a speed of 100 nL/min). Stereotaxic coordinates were based on Paxinos and Franklin's mouse brain atlas (AP=+2.1mm; ML=±0.3mm; DV=-2.2mm)(8). The needle was left in place for an additional 5 min after injection.

Quantitative Reverse Transcriptase PCR. Brains were harvested and snap frozen in isobutane on dry ice and stored at -80°C. Frozen brains were coronally sectioned in a cutting block (Braintree Scientific, Inc., Braintree, MA,) that had been pre-chilled to -20°C. One-millimeter sections were kept frozen throughout dissection with brain regions stored at -80°C until use. Quantitative reverse transcriptase PCR was performed on medial PFC. Total RNA was extracted using the RNeasy Plus Micro Kit (Qiagen, Hilden, Germany). RNA was reverse transcribed using the RevertAid Kit (Thermo Fisher Scientific, Waltham, MA) as per manufacturer's instructions. All Taqman probe sets used were from Thermo Fisher Scientific,

USA: *Reln* (Mm00465200_m1), *Grin1* (Mm00433790_m1), *Grin2a* (Mm00433802_m1), *Grin2b* (Mm00433820_m1) and *Grin2d* (Mm00433822_m1). TaqMan Gene Expression Master Mix from Applied Biosystems (Foster City, CA) was used in generating expression data on a QuantStudio7 thermal cycler. Duplicates were run for each sample, and relative gene expression was determined using the delta delta Ct method standardized against GAPDH expression and normalized to average *Reln* expression in naive mice.

Statistical Analysis. Multiple group comparisons were performed using a non-parametric analysis of variance with a Kruskal-Wallis test. When significant, Dunn's post-hoc tests for multiple comparisons was applied. The effect size Eta-squared (η^2) and P values are provided (9). Paired samples comparisons were conducted using two-tailed Wilcoxon signed-rank tests, and unpaired samples were compared using two-tailed Mann-Whitney test. Distributions were fitted using a nonlinear regression model based on the cumulative Gaussian distribution function, and residuals met normality confirming model assumptions.

Supplementary Figures 1-4

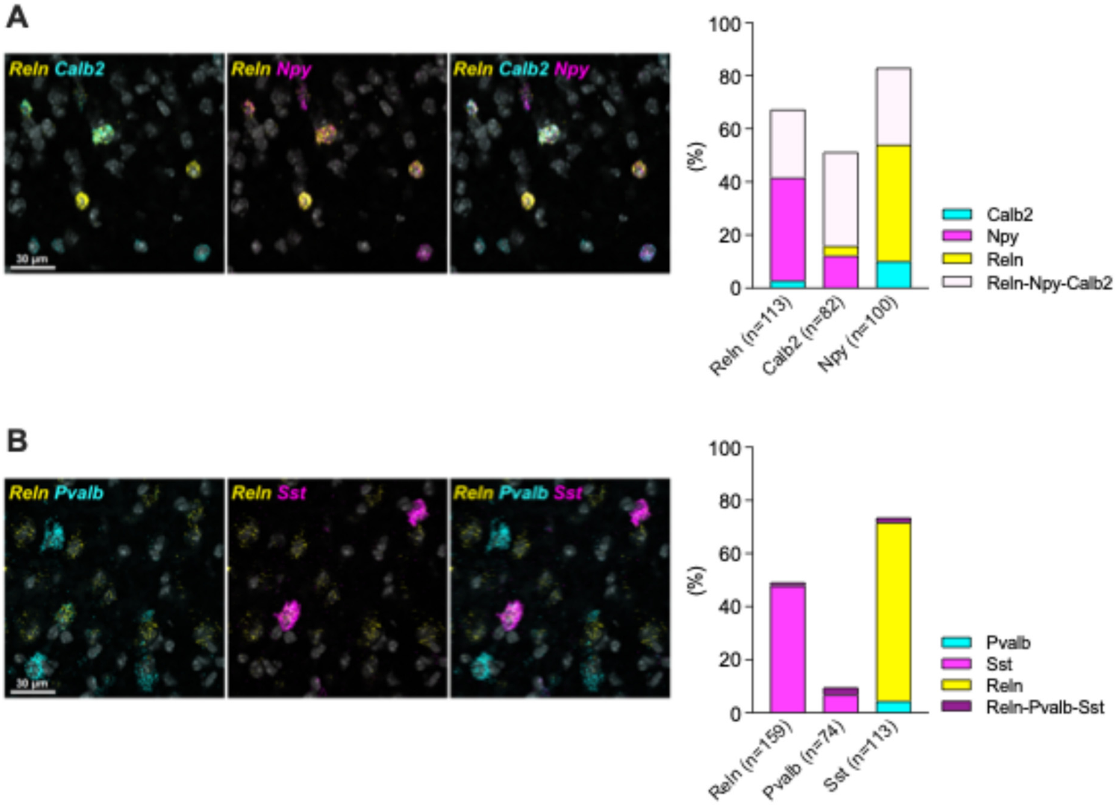

Supplementary Figure 1: Distribution of reelin mRNA in different classes of interneurons.

A: Single-molecular fluorescent in situ hybridization for reelin (*Reln*), calretinin (*Calb2*), and Neuropeptide Y (*Npy*) mRNAs in the in the prelimbic area of the PFC. Slides were counterstained with DAPI (white). Stacked bar graph shows the percentage of cells expressing the different transcript combinations within each neuronal class. **B:** Same legend as A for reelin (*Reln*), parvalbumin (*Pvalb*) and somatostatin (*Sst*). n indicates the total number of cells per neuronal class.

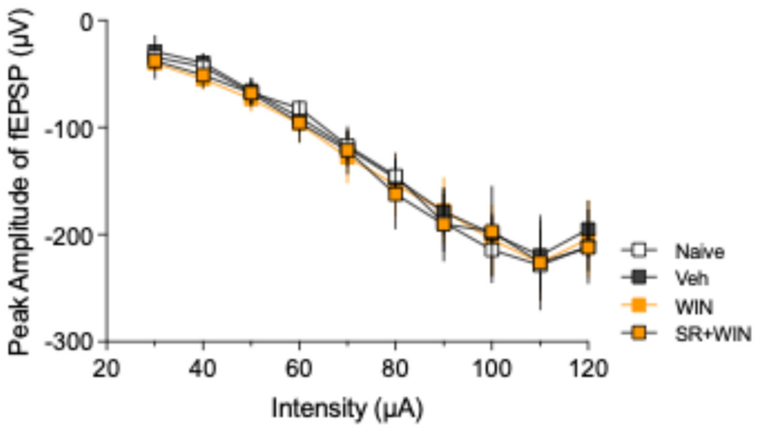

Supplementary Figure 2: Single *in vivo* cannabinoid exposure does not change the input-output profiles from adolescent deep layer PFC synapses.

Input-output curve of average fEPSP amplitude (± sem) is shown for the different groups of mice: naïve (n = 7 mice), vehicle (n = 7 mice), WIN 55,212-2 (WIN, n = 9 mice) and SR141716A + WIN 55,212-2 (SR+WIN, n = 7 mice).

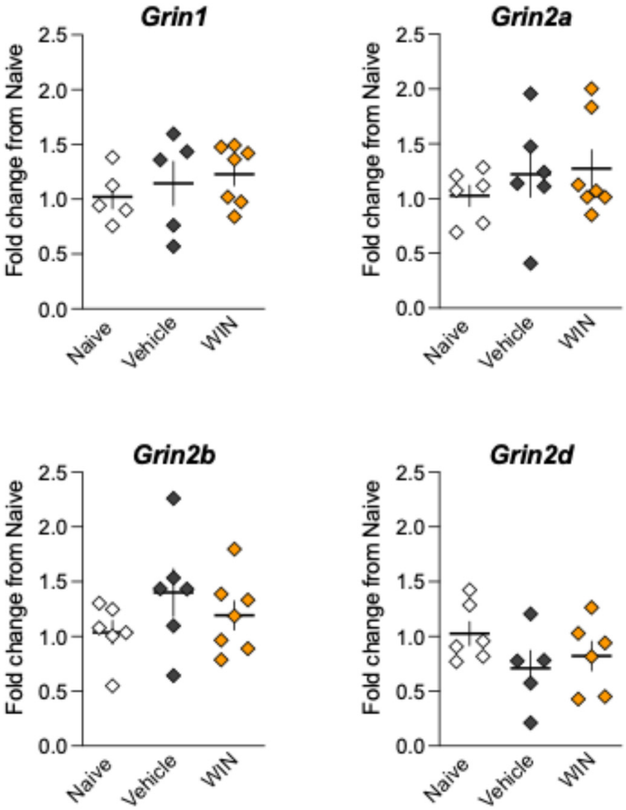

Supplementary Figure 3: Single *in vivo* cannabinoid exposure does not modify mRNA levels of prefrontal NMDAR subunits.

Quantitative polymerase chain reaction analysis of *Grins* mRNA using PFC tissues obtained from the same mice depicted in Figure 3D. mRNA levels of *Grin1* (P = 0.4611, Kruskal-Wallis test), *Grin2a* (P = 0.639, Kruskal-Wallis test), *Grin2b* (P = 0.2377, Kruskal-Wallis test) and *Grin2d* (P = 0.2563, Kruskal-Wallis test). All values are means ± sem and each diamond represents an individual mouse.

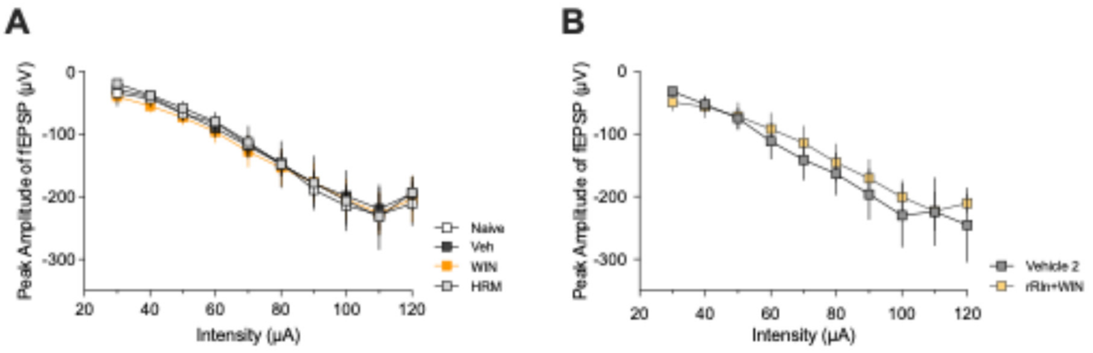

Supplementary Figure 4: Reelin haploinsufficiency and recombinant Reelin supplementation do not change the input-output profiles of adolescent deep-layer PFC synapses.

Input-output relationship of average fEPSP amplitudes (± sem) across stimulus intensities for each group. **A:** Comparaison between HRM (n=9 mice), naïve (n = 7 mice), vehicle (n = 7 mice), and WIN 55,212-2 (WIN, n = 9 mice). **B:** Comparaison between vehicle 2 (n = 3 mice) and recombinant reelin + WIN 55,212-2 (rRln+WIN, n = 5 mice).

References

1. Iafrati J, Orejarena MJ, Lassalle O, Bouamrane L, Gonzalez-Campo C and Chavis P (2014): Reelin, an extracellular matrix protein linked to early onset psychiatric diseases, drives postnatal development of the prefrontal cortex via GluN2B-NMDARs and the mTOR pathway. Mol Psychiatry 19: 417–426.
2. Schindelin J, Arganda-Carreras I, Frise E, Kaynig V, Longair M, Pietzsch T, et al. (2012): Fiji: an open-source platform for biological-image analysis. Nat Methods 9: 676–682.
3. Iafrati J, Malvache A, Gonzalez Campo C, Orejarena MJ, Lassalle O, Bouamrane L, Chavis P (2016): Multivariate synaptic and behavioral profiling reveals new developmental endophenotypes in the prefrontal cortex. Sci Rep 6: 35504.
4. Labouesse MA, Lassalle O, Richetto J, Iafrati J, Weber-Stadlbauer U, Notter T, et al. (2017): Hypervulnerability of the adolescent prefrontal cortex to nutritional stress via reelin deficiency. Mol Psychiatry 22: 961–971.
5. Manduca A, Bara A, Larrieu T, Lassalle O, Joffre C, Layé S, Manzoni OJ (2017): Amplification of mGlu 5 -Endocannabinoid Signaling Rescues Behavioral and Synaptic Deficits in a Mouse Model of Adolescent and Adult Dietary Polyunsaturated Fatty Acid Imbalance. J Neurosci 37: 6851–6868.
6. Borsoi M, Manduca A, Bara A, Lassalle O, Pelissier-Alicot A-L, Manzoni OJ (2019): Sex Differences in the Behavioral and Synaptic Consequences of a Single in vivo Exposure to the Synthetic Cannabimimetic WIN55,212-2 at Puberty and Adulthood. Front Behav Neurosci 13:23.
7. Puighermanal E, Castell L, Esteve-Codina A, Melser S, Kaganovsky K, Zussy C, et al. (2020): Functional and molecular heterogeneity of D2R neurons along dorsal ventral axis in the striatum. Nat Commun 11: 1957.
8. Paxinos G, Franklin KBJ, Franklin KBJ (2001): The Mouse Brain in Stereotaxic Coordinates, 2nd ed. San Diego: Academic Press.
9. Tomczak M. and Tomczak E. The need to report effect size estimates revisited. An overview of some recommended measures of effect size. Trends in Sport Sciences. 2014; 1(21):19-25.
